# Electrochemical Sensing Device for Carboplatin Monitoring in Proof-of-Concept Drug Delivery Nanosystems

**DOI:** 10.3390/nano14090793

**Published:** 2024-05-02

**Authors:** Alexandra Pusta, Mihaela Tertis, Catalina Ardusadan, Simona Mirel, Cecilia Cristea

**Affiliations:** 1 Department of Analytical Chemistry, Iuliu Hatieganu University of Medicine and Pharmacy, 400349 Cluj-Napoca, Romania; alexandra.pusta@umfcluj.ro (A.P.); catalina.ralu.ardusadan@elearn.umfcluj.ro (C.A.); ccristea@umfcluj.ro (C.C.); 2 Department of Medical Devices, Iuliu Hatieganu University of Medicine and Pharmacy, 400349 Cluj-Napoca, Romania; smirel@umfcluj.ro

**Keywords:** carboplatin, nanosomes, in-house screen-printed electrodes, electrochemical detection

## Abstract

(1) Background: Carboplatin (CBP) is a chemotherapeutic drug widely used in the treatment of a variety of cancers. Despite its efficiency, CBP is associated with side effects that greatly limit its clinical use. To mitigate these effects, CBP can be encapsulated in targeted delivery systems, such as liposomes. Ensuring the adequate loading and release of CBP from these carriers requires strict control in pharmaceutical formulation development, demanding modern, rapid, and robust analytical methods. The aim of this study was the development of a sensor for the fast and accurate quantification of CBP and its application on proof-of-concept CBP-loaded nanosomes. (2) Methods: Screen-printed electrodes were obtained in-lab and the electrochemical behavior of CBP was tested on the obtained electrodes. (3) Results: The in-lab screen-printed electrodes demonstrated superior properties compared to commercial ones. The novel sensors demonstrated accurate detection of CBP on a dynamic range from 5 to 500 μg/mL (13.5–1350 μM). The method was successfully applied on CBP loaded and released from nanosomes, with strong correlations with a spectrophotometric method used as control. (4) Conclusions: This study demonstrates the viability of electrochemical techniques as alternative options during the initial phases of pharmaceutical formulation development.

## 1. Introduction

Cancer therapy is one of the mainstays of medical research, due to the increasing incidence of the disease, but also thanks to technological advances that create new possibilities for treatment. Different strategies for cancer therapy have been developed in recent years, such as vaccines [1], immunotherapy [2], intestine-inspired delivery systems [3], flexible electrohydrodynamic pumps [4], and targeted drug delivery systems such as liposomes, dendrimers, metallic particles, polymeric particles, or others [5]. The latter aims at reducing the systemic side effects of chemotherapeutic drugs, by delivering them strictly in the proximity of the tumor. This could lead to improved safety profiles and better patient compliance. Although liposomal formulations containing certain drugs, such as doxorubicin, are already present on the market [6], there remains a lack of commercial availability for other drugs in this particular form.

One such drug is carboplatin (CBP), a second-generation platinum-based chemotherapeutic used in the treatment of advanced ovarian cancer [7], small cell lung carcinoma [8], and head and neck cancers [9]. CBP is known for its systemic side effects such as myelosupression, nephrotoxicity, ototoxicity, cardiotoxicity, and peripheral neurotoxicity [10]. The onset of these side effects could be postponed or eliminated by the encapsulation of CBP in drug delivery systems such as PEGylated niosomes [11], bovine serum albumin nanoparticles [12], gelatin nanoparticles [13], chitosan nanoparticles [14], graphitic carbon nitride [15], or polymer-functionalized metal-organic frameworks [16].

Given the ongoing development of numerous CBP formulations, it is essential that analytical methods for the quantification of CBP advance in tandem. The accurate quantification of CBP is crucial to guarantee optimal loading and release profiles in/from these formulations, especially in the initial phases of pharmaceutical formulation development, where numerous tests need to be performed, this being the main objective of this study.

Apart from pharmaceutical formulation development, the quantification of CBP is also important in the clinical setting. The detection and monitoring of CBP concentrations in biological fluids can help ensure treatment safety and prevent the onset of side effects [17].

Different methods, such as liquid chromatography [18], liquid chromatography coupled with tandem mass spectrometry [19,20], micellar and microemulsion electrokinetic chromatography [21], capillary zone electrophoresis [22], and fluorescence after derivatization [23], have been employed for CBP detection in pharmaceutical formulations or biological fluids. Although these methods offer high accuracy, they often require substantial sample volumes, which may not be readily accessible during the initial phases of pharmaceutical formulation development. Furthermore, they entail significant costs and require specialized personnel.

An alternative to these is represented by electrochemical methods. They are accurate, fast, portable and, once optimized, can be used with minimum training [24]. Among electrochemical methods, voltammetric techniques are commonly used for the direct detection of small molecules, due to their high sensitivity. They involve scanning the potential and measuring the current generated by the oxidation/reduction in the analyte. A disadvantage of voltammetric methods is electrode fouling and surface contamination [25]. To mitigate this, single-use electrodes can be developed through a variety of techniques, such as screen printing. Screen printing involves the deposition of conductive inks with different compositions (carbon, gold, silver) onto plastic or ceramic substrates to produce electrodes that present advantages such as flexibility, reproducibility, ease of manufacture, ease of surface functionalization, low costs, and capacity for mass production [26].

The direct electrochemical detection of CBP was performed by using platinum [27,28] or carbon paste electrodes [28]. Indirect detection approaches consisted in the detection of CBP using DNA-modified glassy carbon electrodes [29] or carbon nanotube-epoxy composite electrodes in the presence of adenine or guanine [30]. In these approaches, the electrochemical detection was carried out by following the changes in the oxidation signal of DNA bases in the presence of CBP.

In this work, the direct electrochemical detection of CBP based on its electrochemical oxidation signal obtained on in-lab screen-printed carbon electrodes is reported for the first time. The electrodes were screen-printed using conductive carbon and silver inks and the electrochemical behavior of CBP was analyzed on the in-house screen-printed surface. The influence of the pH and the scan rate were identified and a differential pulse voltammetry (DPV) method was optimized for CBP detection. The second objective of this study was the application of the developed method for the analysis of CBP-containing pharmaceutical formulations. To this scope, nanosomes were loaded with CBP, followed by the release of the drug in different conditions. The developed electrochemical method was applied for the quantification of the loaded/released CBP and the results were in good correlation with the UV–Vis spectrophotometric method used as control. The optimized DPV method was also applied for the detection of CBP from commercial artificial saliva, artificial tears, and commercial human serum, which were used as model complex matrices to expand the practical application of the analytical method and the developed sensor. The robustness of the DPV method was evaluated by ANOVA statistical analysis to assess the CBP concentration in real samples, serving as intra-assay validation for the optimized method.

The present work demonstrates the potential applicability of electrochemical methods both in the early stages of pharmaceutical formulation development and in the detection of CBP in biological complex matrices for diagnosis and treatment monitoring. The in-lab screen-printed electrodes demonstrated better performances compared to commercial ones, while also presenting lower manufacturing costs. The analysis time is short and requires low sample volumes, compared to other methods described in the literature such as liquid chromatography. Moreover, the direct electrochemical oxidation of CBP is reported, thus avoiding the need for unstable reagents such as DNA or DNA bases for indirect electrochemical detection.

## 2. Materials and Methods

### 2.1. Materials

CBP was purchased from Santa Cruz Biotechnology (Dallas, TX, USA). 23.5 mg/mL CBP stock solutions were prepared in 0.1 M H_2_SO_4_, aliquoted and stored at −20 °C for maximum one month. Lipo-N type nanosomes (100 nm) were purchased from Nanovex Biotechnologies (Asturia, Spain) and used for CBP loading and release studies. All other reagents were purchased from Sigma Aldrich (Saint Louis, MO, USA), were of analytical grade, and were used without further purification. All solutions were prepared in ultrapure water (18 MΩ cm^−1^) obtained using a Millipore Simplicity device (Sigma Aldrich, Saint Louis, MO, USA).

### 2.2. Methods

#### 2.2.1. Electrochemical Cell Printing and Characterization

The electrochemical cells were fabricated according to a previously published procedure [31]. A stainless-steel stencil was utilized to print the contacts and electrodes. Initially, an Electrodag PF-410 silver conductive ink (Henkel, Dusseldorf, Germany) was employed to print the contacts and reference electrodes. The printed silver layer was then allowed to dry for 15 min at 60 °C. Subsequently, the working and counter electrodes were printed using an Electrodag 423 SS© carbon-based ink (Henkel, Dusseldorf, Germany), followed by drying at 60 °C for 15 min. Finally, an insulating layer was applied to prevent short circuits, and the electrochemical cells were left to dry overnight at 60 °C to ensure complete polymerization of the ink monomers and thorough drying. After imprinting, the electrodes were electrochemically pre-treated using amperometry at a constant potential of +1.2 V for 600 s in a 1 M Na_2_CO_3_ solution.

The electrode surface underwent electrochemical characterization after printing, employing electrochemical impedance spectroscopy (EIS) and cyclic voltammetry (CV) in a 10 mM [Fe(CN)_6_]^3−/4−^ solution prepared in 0.1 M KCl. EIS was conducted over 61 frequencies ranging from 0.1 to 100,000 Hz with an amplitude of 0.01 Hz at the open-circuit potential. CV involved scanning the potential between −0.4 and +1 V for 2 cycles at a scan rate of 100 mV/s.

#### 2.2.2. Electrochemical Measurements

An Autolab MAC80100 multichannel potentiostat/galvanostat (Metrohm, Utrecht, The Netherlands) operated with Nova 1.10.4 software was used to perform the electrochemical tests. Commercial carbon electrodes (Metrohm DropSens, Oviedo, Spain) and in-house screen-printed carbon electrodes were used to analyze the electrochemical behavior of CBP and the best surface was chosen for further tests. To analyze the influence of the pH on the detection of CBP, 100 μg/mL CBP solutions were prepared in Britton–Robinson buffer (BRB) with pH values ranging from 2 to 10. The solutions were tested using a CV procedure as follows: scan between 0.2 and +1.4 V, scan rate 100 mV/s.

The influence of the scan rate was determined using 100 μg/mL CBP by CV by cycling the potential between 0.2 and +1.5 V with scan rates between 5 and 500 mV/s.

The influence of CBP concentration on the analytical signal was determined using DPV on a concentration domain between 5 and 500 μg/mL, on lab-made screen-printed electrodes. The optimized DPV conditions were as follows: scan between 0 and +1 V with a scan rate of 100 mV/s and a step potential of 5 mV.

The selectivity of the method was tested using the optimized DPV method on CBP solutions in the presence of common interferents present in pharmaceutical formulations or biological fluids, such as dopamine, ascorbic acid, citrate, Ca(II), Na(I), Mg(II), Li(I), NH_4_(I), Cl^−^, Br^−^, SO_4_^2−^, NO^3−^.

For real sample analysis, artificial saliva (Sigma Aldrich, St Louis, MO, USA) and artificial tears (Systane^®^ ULTRA, Alcon^®^, Geneva, Switzerland) were diluted and spiked with appropriate concentrations of CBP. In the case of serum samples, a 4 M ammonium sulfate solution was used for protein precipitation from commercial human serum (Sigma Aldrich, Saint Louis, MO, USA). The samples were centrifuged for 5 min at 10,000 rpm and the obtained supernatant was diluted ten times with PBS and then spiked with CBP. The concentration of the spiked samples was determined using the calibration curve. The recoveries and relative standard deviations were calculated for all measurements and the robustness of the assay was validated using ANOVA statistical analysis.

For the analysis of proof-of concept pharmaceutical formulations, Lipo-N nanosomes were loaded with CBP and the amount of loaded CBP was determined electrochemically with the optimized DPV method, as well as by UV–Vis spectrophotometry used as a control method. The release of CBP from the nanosomes was monitored using the same techniques.

#### 2.2.3. Carboplatin Loading

A total of 100 mg of nanosomes were accurately weighted in Eppendorf tubes and a 2 mg/mL CBP solution prepared in 0.1 M phosphate-buffered saline (PBS) pH 5 was put in contact with the nanosomes for 24 h at room temperature. The suspensions were placed on a HulaMixer (Invitrogen) under continuous shaking, using the following parameters: orbital shaking 3 rpm (90 s), reciprocal shaking 1° (10 s), and vibration movement 1° (10 s). After 24 h, the suspensions were centrifuged for 10 min at 12,000 rpm and 350 μL of the supernatant was sampled and tested using either electrochemical or spectrophotometric methods. For the electrochemical method, a 1:1 dilution with 0.1 M PBS pH 5 was performed before testing, while for the spectrophotometric method no dilution was required. The CBP concentration was determined using the corresponding calibration curves and the encapsulation efficiency (EE%) and loading capacity (LC%) were calculated using the following equations [32]:

EE (%) = ((V × C_i_ − V × C_f_)/(V × C_i_) × 100);

LC (%) = ((V × C_i_ − V × C_f_)/m_nanosomes-CBP_ × 100)

where V—volume of the release media, C_i_—initial CBP concentration, C_f_—final CBP concentration (after loading), and m_MCPs-carboplatin_—weight of the loaded nanosomes.

#### 2.2.4. Carboplatin Release

A total of 100 mg of loaded nanosomes was accurately weighted in Eppendorf tubes and suspended in 0.1 M PBS pH 5, pH 6, and pH 7.4, respectively. The release experiments were performed at 37 °C for three days, under continuous shaking in a ThermoMixer (Eppendorf®, Hamburg, Germany). The samples were centrifuged to separate the nanosomes from the supernatant and 350 μL of supernatant was sampled at precise time intervals, as follows: at 15 min intervals in the first hour, at 1 h intervals in the first 6 h, and then at 24 h intervals for three days. After each sampling, 350 μL of the corresponding fresh buffer was added to the samples. All release experiments were performed in triplicate.

CBP concentration in the release media was determined using both the spectrophotometric method and the optimized electrochemical method. The cumulative mass of released CBP and the cumulative release was determined using the following equations [32]:

m_n_ = (C_n_ × V) + V_s_ (C_1_ + C_2_ + C_3_ +…+ C_n−1_)

Cr_n_ = (m_n/_m_load_ × 100)

where m_n_—cumulative mass at a certain time point, C_n_—CBP concentration in the release media at a certain time point, V—volume of the release media, V_s_—volume sampled for analysis (350 μL), C_1,2,…n−1_—CBP concentration at previous testing times, Cr_n_—cumulative release, m_n_—cumulative mass, m_load—_mass of CBP loaded in the nanosomes.

#### 2.2.5. Spectrophotometric Measurements

UV–Vis spectrophotometric measurements were conducted utilizing a SPECORD 250PLUS spectrophotometer (Analytik Jena, Jena, Germany) controlled by the WinAspect 4.2.0.0. software. Various concentrations of CBP were dissolved in different buffers, and their spectra were recorded within the range of 200–500 nm. The wavelength corresponding to maximum absorbance (λ_max_) was determined, and calibration curves were built for CBP in each buffer tested. These curves were then used to quantify the amount of CBP loaded into and released from the nanosomes.

#### 2.2.6. Statistical Analysis

ANOVA statistical analysis was performed using the Excel 2016 software.

## 3. Results and Discussions

### 3.1. Electrochemical Cell Development

**Figure 1:**
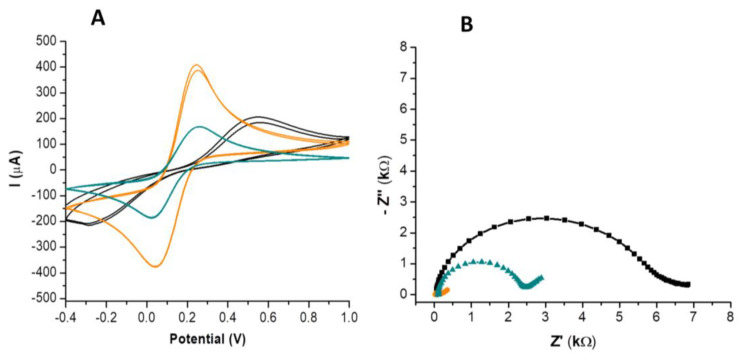
Surface characterization in 10 mM [Fe(CN)_6_]^3^−^/4^− via CV for the commercially available carbon electrode (green, in-lab screen-printed carbon electrode (black), activated in-lab screen-printed carbon electrode (orange) (**A**); Surface characterization in 10 mM [Fe(CN)_6_]^3^−^/4^−via EIS for the commercially available carbon electrode (green), in-lab screen-printed carbon electrode (black), activated in-lab screen-printed carbon electrode (orange) (**B**).

**Figure 2:**
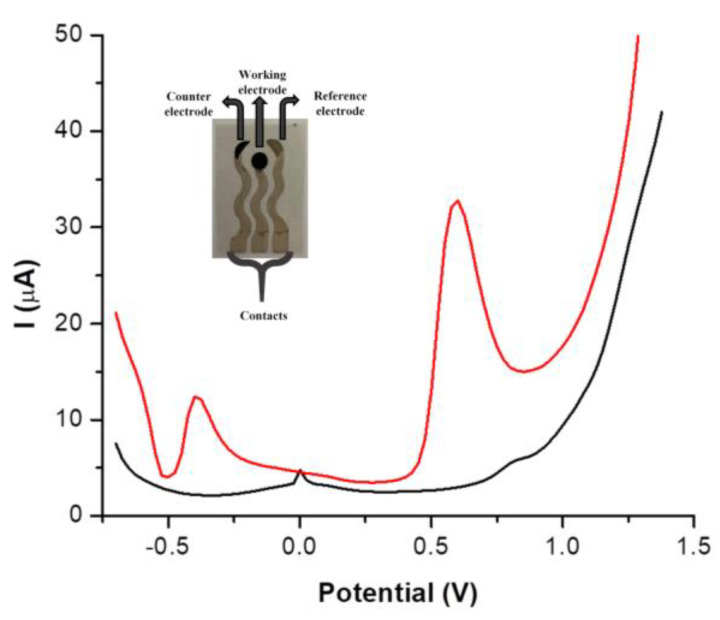
Differential pulse voltammograms indicating the electrochemical behavior of CBP on the in-house screen-printed electrode (red) compared to a blank solution—0.1 M PBS pH 5 (black). **Inset**: Image of the in-lab screen-printed electrochemical cell.

The in-house screen-printed carbon electrochemical cell was developed, and its performance was compared to that of commercially available carbon screen-printed electrodes (results presented in Table 1 and Figure 1). The comparison was performed using CV and EIS in a 10 mM [Fe(CN)_6_]^3−/4−^ solution prepared in 0.1 M KCl solution. The in-house screen-printed cell consisted of silver contacts, a carbon working and counter electrode, and a silver reference (see Inset in Figure 2). The diameter of the working electrode was 4 mm and the height of the screen-printed surface was 0.15 mm. The dimensions of the whole electrochemical cell, including contacts, were 5 × 1 cm (length vs. width).

It can be observed in Table 1 that there were no significant differences between the oxidation and reduction current intensities obtained on the commercial and the in-house screen-printed carbon surfaces. However, in the case of the in-house screen-printed electrode, a peak-to-peak separation of about 800 mV was registered, compared to just 200 mV for the commercial electrode (Table 1, Figure 1A). This prompted the use of a surface activation procedure already described in the literature [31], consisting of amperometry at +1.2 V for 600 s in a 1 M Na_2_CO_3_ solution. After the pretreatment procedure, both the oxidation and reduction signals increased by almost 100%, while the peak-to-peak separation decreased to 200 mV, as in the case of the commercially screen-printed electrode (Table 1, Figure 1A). The data obtained by CV were also confirmed by EIS analysis (Figure 1B). It can be observed that the highest resistance to charge transfer was registered in the case of the in-house screen-printed, inactivated electrode (R_ct_ = 5744 Ω). After activation, a sharp decrease to 147 Ω was observed. In the case of the commercial electrode, the registered R_ct_ was 2227 Ω, indicating that the activated in-house screen-printed electrode presents a better surface conductivity compared to the commercial one. This demonstrates that adequately pretreated in-house obtained electrodes can be successfully used as an alternative to commercially screen-printed electrodes, offering higher current intensities with the same peak-to-peak separation.

The electroactive area of the in-house screen-printed electrodes was calculated based on the analysis of [Fe(CN)_6_]^3−/4−^ behavior and the Randles–Sevcik equation [33]:

$$I_{p}=(2.69\cdot 10^{5})\cdot n\cdot \sqrt{\alpha \cdot n_{\alpha }}\cdot C\cdot \sqrt{D}\cdot \sqrt{v}\cdot S$$

where *I_p_*—intensity of the peak current; *n*—number of electrons involved in the electrochemical process; *α*—transfer coefficient; *C*—concentration of the redox species in mol/cm^3^; *D*—diffusion coefficient in cm^2^/s; *v*—scan rate (V/s); *S*—surface area of the working electrode (cm^2^).

The calculated electroactive area was *S* = 0.308 cm^2^, which explains the differences obtained between the in-house screen-printed sensor and the commercially available one, which has an electroactive area of *S* = 0.126 cm^2^. The difference in area leads to the differences observed in Table 1 and Figure 1.

### 3.2. Electrochemical Behavior of CBP

The electrochemical behavior of CBP was analyzed on the in-house screen-printed electrodes and in DPV two oxidation peaks were observed at around −0.375 V and +0.6 V (Figure 2). The oxidation of cisplatin, another platinum-based chemotherapeutic, was previously reported in the literature at negative potentials around −0.5 V [34]. Despite this, in this work the oxidation of CBP was followed at +0.6 V, due to the higher intensity of the oxidation signal at this potential, this having an approximately threefold higher intensity compared to the signal at −0.375 V.

**Figure 3:**
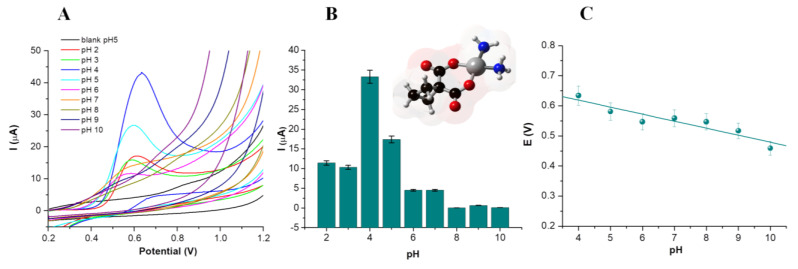
The voltammograms obtained on the activated in-house screen-printed electrodes for CBP 100 µg/mL in BRB solutions with pH 2–10 (**A**). Variation in the current intensity with the pH on the 2–10 pH range (**inset**: structure of CBP) (**B**). Variation in the oxidation potential of CBP with the pH of the supporting buffer (**C**).

**Figure 4:**
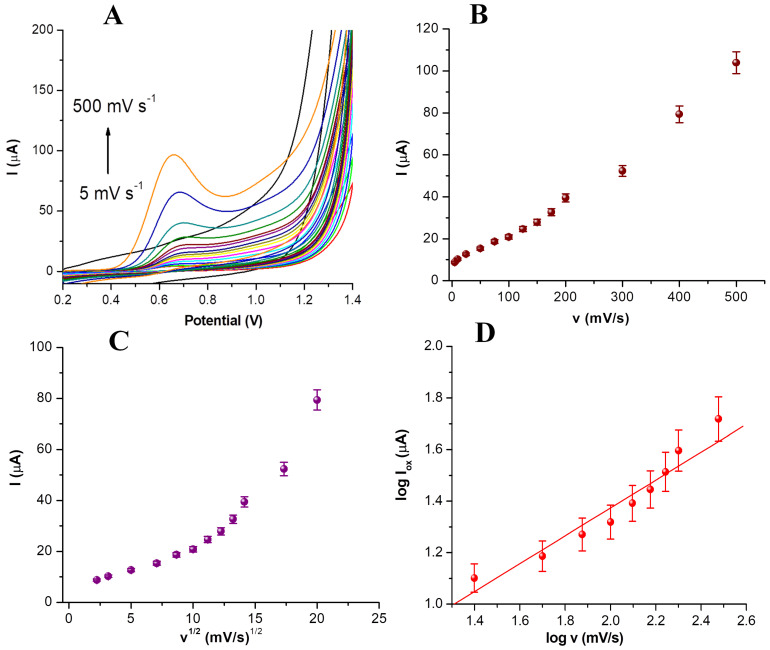
Cyclic voltammograms registered for the 100 μg/mL CBP solution between +0.2 and +1.5 V with scan rates between 5 and 500 mV/s in 0.1 M PBS pH 5 (**A**); Variation in the intensity of the oxidation current for CBP with the scan rate (**B**); Variation in the intensity of the oxidation current for CBP with the square root of the scan rate (**C**); Variation in the logarithm of the current intensity for CBP with the logarithm of the scan rate (**D**).

The oxidation of CBP on the in-house screen-printed electrodes was irreversible (see Figure 3 and Figure 4).

#### 3.2.1. Influence of the pH

To study the influence of the pH on the electrochemical behavior of CBP, 100 µg/mL solutions were prepared in BRB with pH values between 2 and 10. The obtained voltammograms are presented in Figure 3. It can be noticed that, in all cases, the oxidation of CBP is irreversible, which is consistent with previous data published in the literature [27]. A cathodic shift in the oxidation peak can be observed with the increase in the pH (Figure 3A). This can be explained by the involvement of protons in the oxidation mechanism of CBP [28]. The intensity of the oxidation peak increases on the pH 2–4 domain, registering a maximum value at pH 4, followed by a decrease to pH 5 and a further decrease to a plateau that remains relatively constant up to pH 10 (Figure 3B). This behavior also suggests the involvement of protons in the oxidation mechanism of CBP. A possible oxidation mechanism for CBP was proposed by Mebsout et al. [28] and suggests the addition of H_2_O or Cl^−^ ligands to the oxidized platinum (IV) center. The type of ligand is determined by the Cl^−^ concentration of the buffer used. In this case, Cl^−^ ions were absent from the Britton–Robinson buffer, which determines that the most likely structure for the oxidation product is the one with water molecules as ligands. A similar mechanism was proposed for the electro-oxidation of cisplatin, a chemotherapeutic similar in structure to CBP [34].

While the maximum current intensity was observed at pH 4, the pH selected for further tests was 5, given its proximity to the pH of the tumor tissue and the maintenance of a sufficiently high current intensity.

The variation in the oxidation potential with the pH is represented in Figure 3C and proved to be linear on the pH domain between 4 and 10. The correlation was expressed by the equation E(V) = −0.023 × pH + 0.712 and had a correlation coefficient of 0.97. The slope obtained for this equation corresponds to a number of two electrons, confirming the mechanism proposed in the literature for the electrochemical transformation of CBP [28].

#### 3.2.2. Influence of the Scan Rate

Considering the previously presented results, the influence of the scan rate on the electrochemical oxidation of CBP was tested in 0.1 M PBS pH 5. This medium was chosen to assess the behavior of CBP in the environment in which the release of the drug will be tested. The results obtained in CV for scan rates between 5 and 500 mV/s are presented in Figure 4A.

The increase in the scan rate led to an increase in the oxidation current intensity as well as to a slight cathodic shift (Figure 4A). A scan rate of 100 mV/s was chosen for further experiments as this value provided a high enough current intensity and was slow enough to capture the electrochemical transformation of CBP. The variation of peak current intensity with the scan rate (Figure 4B) and with the square root of the scan rate (Figure 4C) were represented to determine whether the electrochemical process was diffusion or adsorption-controlled.

For the variation in the peak current intensity with the scan rate, the following equation was obtained: I_ox_ (µA) = 0.185v (mV/s) + 4.350, while for the variation in the peak current intensity with the square root of the scan rate, the equation was I_ox_ (µA) = 4.29v^1/2^ (mV/s)^1/2^ − 14. It can be seen that a better linear correlation was obtained for the variation of the current intensity with the scan rate (R^2^ = 0.980), than with the square root of the scan rate (R^2^ = 0.862), indicating an adsorption-controlled process. This is in contradiction with previously published results, which identified a diffusion-controlled behavior of CBP on platinum [27] or carbon paste electrodes [28]. To better understand the kinetics of the electro-oxidation process, the variation in the logarithm of the current intensity with the logarithm of the scan rate was represented for CBP in 0.1 M PBS pH 5 (Figure 4D). A linear correlation (log(I_ox_ (µA)) = 0.52 log (v (mV/s)) − 0.40 with a slope coefficient of 0.52 was obtained, which is close to the theoretical value of 0.5 for diffusion-controlled processes. This is in agreement with previously published data [27,28] and indicates that, overall, the electro-oxidation process in this case is controlled by both diffusional and adsorptive processes. This phenomenon may be due to the nature of the in-lab screen-printed electrode surface, which may present roughness and irregularities.

### 3.3. Analytical Parameters

**Figure 5:**
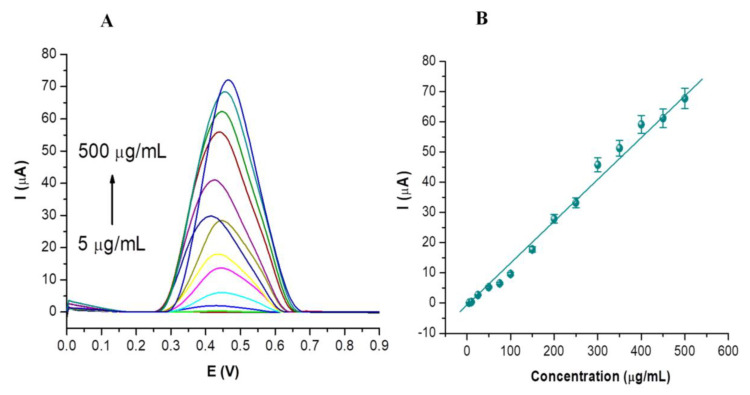
Voltammograms obtained following the testing of CBP solutions at various concentrations (5, 10, 25, 50, 75, 100, 150, 200, 250, 300, 350, 400, 450, 500 µg/mL) prepared in 0.1 M PBS at pH 5 over the potential range from 0 V to 1 V (**A**) Calibration curve obtained for the tested CBP concentrations (**B**).

Based on the previous results, an optimized method was developed for the detection of CBP based on DPV, using the following parameters: scan rate 100 mV/s, 0.1 M PBS pH 5 as electrolyte media, potential scan between 0 and +1 V. The voltammograms obtained for increasing concentrations of CBP between 5 and 500 µg/mL are presented in Figure 5A. A linear variation was observed between the oxidation current intensity of CBP and its concentration across the entire tested range (Figure 5B).

For the linear variation between the peak current and concentration, an equation of the form I_ox_ (µA) = 0.145 [CBP] (µg/mL) − 1.946 was obtained, displaying a correlation coefficient R^2^ = 0.9914. All tests were performed in triplicate and the average standard deviation was ±1.69%. A detection limit (LOD) value of 1.6 µg/mL was estimated based on the signal-to-noise (S/N) ratio of 3, and a limit of quantification (LOQ) of 5 µg/mL was established, representing the lower limit of the concentration range of CBP tested in this study. The sensitivity of the optimized DPV method for CBP detection is 0.145 µA mL/µg, which was determined based on the slope of the calibration curve.

The inter-electrode variability was tested on five different electrodes produced in different batches and the calculated RSD was 4.15%.

The obtained results were compared with data from the literature and presented in Table 2 (results from this work written in bold).

Compared with other direct electrochemical detection methods, this study presents the lowest LOD and the widest linear range. Moreover, the LOD is comparable to that obtained for an indirect detection strategy that was also applied on human serum samples [29]. However, the indirect method relies on DNA for electrode functionalization, which could lead to potential stability and reproducibility issues. The detection of CBP was achieved by Phairatana et al. [30], using a microfluidic system which integrated a glassy carbon electrode modified with a carbon nanotube-epoxy composite. While this allows for CBP detection at very low concentrations, the experimental setup is complex, and the detection of CBP is also indirect, based on changes in the electro-oxidation signal of adenine. The obtained results indicate that the present method represents a simple, fast, and cost-efficient strategy for CBP detection.

### 3.4. Selectivity Studies

The detection of CBP was performed in the presence of different possible interferents from pharmaceutical formulations (Ca(II), Na(I), Mg(II), Cl^−^), biological fluids (dopamine, ascorbic acid), loading and release media. The tested interferents were chosen based on a literature study, which proposed these compounds as possible interferents in the electrochemical detection of cisplatin, a drug with structural similarities with CBP [35]. Single-component solutions of varying concentrations of the following compounds were tested: dopamine, ascorbic acid, citrate, Ca(II), Na(I), Mg(II), Li(I), NH_4_(I), Cl^−^, Br^−^, SO_4_^2−^, NO^3−^, as well as mixtures of CBP with the aforementioned compounds at equal concentrations. The results were compared with the peak oxidation current values obtained for CBP from single-component standard solutions of the same concentration. The tested concentration values and the recovery rates calculated for the CBP signal in the presence of interferents compared to the signal obtained when it is alone in solution are presented in Table 3. All tests were performed in triplicate and the relative standard deviations (RSDs) are presented in Table 3.

No interferences were observed in the case of dopamine, ascorbic acid, Ca(NO_3_)_2,_ sodium citrate, MgCl_2_, Li_2_SO_4,_ and NH_4_Cl at all tested concentrations, with acceptable recovery rates ranging from 87.81% to 111.72%. Slight interferences could be observed in the case of lower concentrations of MgBr_2_. The electrochemical analysis of cisplatin (CIS), a similar platinum-based chemotherapeutic agent, led to the formation of an oxidation peak at a higher potential compared to that for the oxidation of CBP. When the mixture of the two compounds was tested, the oxidation peaks merged into one, making the detection of CBP in the presence of CIS impossible. However, this interference does not present clinical relevance, as the two compounds are not administered together, being representatives of the same chemotherapeutic class. In general, associations of multiple drugs from the same class are avoided, due to the increased risk for synergic negative effects, with no improvement in therapeutic efficiency.

### 3.5. Real Sample Analysis

The detection of CBP in biological fluids is justified as the optimized method developed in this study could be implemented as a monitoring technique during chemotherapy to assess treatment safety. Plasma concentrations of CBP are used in clinical practice to evaluate clinical exposure to the drug and determine the right dosage, especially in vulnerable populations such as neonates [36]. Apart from serum or plasma samples, less invasive biological fluids such as tears, saliva, or sweat, could also be employed, considering patient non-compliance with invasive serum sampling methods.

Commercial serum samples were treated with saturated ammonium sulfate solution for protein precipitation and were diluted ten-fold with PBS to dilute the precipitation agent. Artificial saliva and tears were also used as model complex matrices for the detection of CBP, as the presence and persistence of CBP in saliva up to 24 h after administration was demonstrated in previous studies [37].

Deproteinized serum, commercial saliva, and tear samples enriched with CBP were tested in the study, for which recovery rates and RSD for the current signal were calculated relative to the calibration curve data. The results are presented in Table 4, showing no significant matrix effect for samples reconstituted in deproteinized serum, saliva, and artificial tears at a 1:10 dilution. These results indicate the applicability of the method for the detection of CBP from model biological matrices after minimal pretreatment.

#### 3.5.1. Evaluation of the Robustness of the Applied Electrochemical Method for CBP Detection in Serum, Saliva, and Tears

Evaluation of robustness of the applied DPV method was assessed using ANOVA statistical analysis to determine the amount of CBP found in real samples such as deproteinized serum, tears, and saliva (the intra-assay validation of the optimized method). Recovery values were determined at three different concentrations, each with three replicates, and the ANOVA statistical analysis revealed favorable robustness results, as depicted in Table 5. Regression analysis across all experiments demonstrated high significance, with no deviations observed in either parallelism or linearity (*p* = 0.279 > p_theoretical_ = 0.05). Furthermore, all assays yielded results within the confidence interval, indicating a proper execution of the assay system. Additionally, the optimized method exhibited significant response differentiation between concentrations and notable sensitivity to the selected concentrations, as summarized in Table 5.

#### 3.5.2. Comparison between DPV and UV–Vis Methods

To compare the DPV method proposed in this study with the UV–Vis method, both techniques were utilized for the analysis of human deproteinized serum, saliva, and tears containing CBP concentrations ranging from 25 to 200 μg/mL (with three replicates for each concentration). The *t*-test (two-sample assuming equal variances) was also conducted, revealing no significant difference between the datasets (*p* = 0.211 > p_theoretical_ = 0.05). The correlation between the CBP concentrations determined by the DPV assay and those obtained from the UV–Vis assay in real samples was then assessed.

### 3.6. Spectrophotometric Studies

UV–Vis spectrophotometry was used as a control method to verify the results obtained from DPV studies for the loading and release of CBP. The spectrophotometric behavior of CBP was tested in all buffers that were used in this study, the wavelength corresponding to the maximum of absorption was determined in each case, and a calibration curve was built using increasing concentrations of CBP dissolved in each buffer. The obtained parameters are presented in Table 6.

### 3.7. Application of the Electrochemical Method for the Characterization of Pharmaceutical Formulations

#### 3.7.1. Evaluation of the CBP Loading Process

CBP loading was performed from 2 mg/mL solutions prepared in 0.1 M PBS pH 5. Table 7 shows the EE% and LC% values obtained using the concentration of CBP determined in the supernatant using the UV–Vis and the DPV methods, respectively.

The variability between the two methods was calculated and a ±2.38% variability was obtained for EE% and ±1.76% for LC. The low variability between the two methods indicates that the DPV method can be successfully used for loading studies.

#### 3.7.2. Evaluation of the CBP Release Process

**Figure 6:**
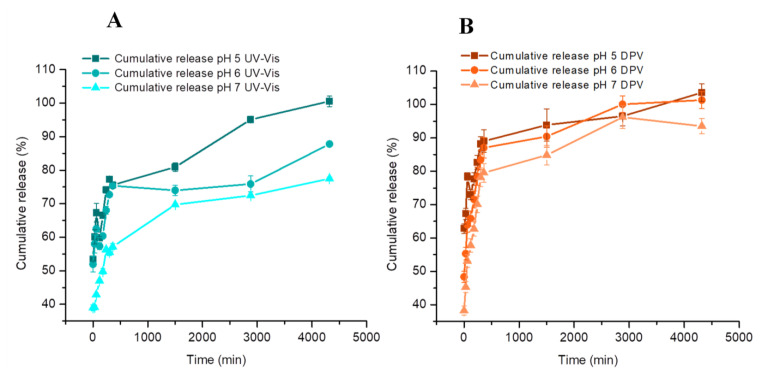
The representation of the cumulative release variation for CBP from nanosomes in 0.1 M PBS with pH 5, 6, and 7.4, using the optimized DPV procedure and the electrochemical activated graphite-based sensor (**A**), respectively, through UV–Vis spectrophotometry (**B**). Error bars represent the standard deviation of three tests.

**Figure 7:**
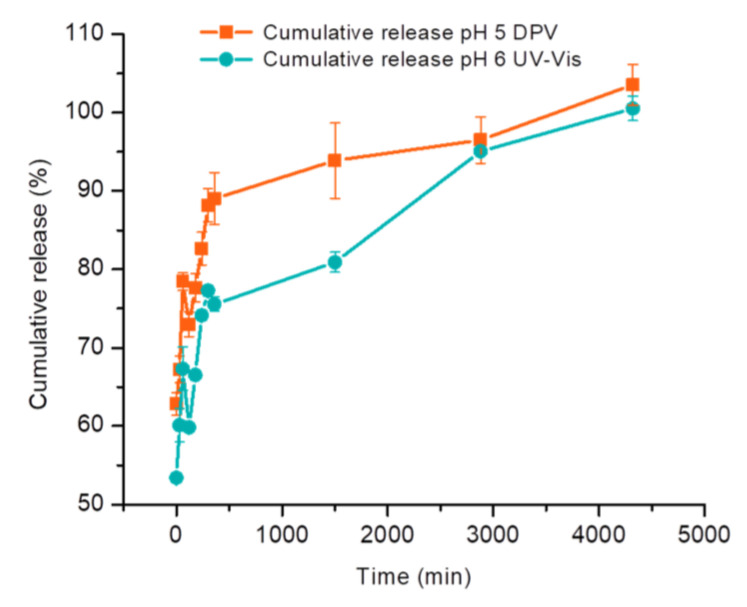
Comparison between the cumulative release obtained using the optimized electrochemical method (■) and UV–Vis spectrophotometry (●) recorded for the release of CBP from nanosomes loaded from a 2 mg/mL CBP solution prepared in 0.1 M PBS pH 5. The release was carried out in 0.1 M PBS pH 5 and tested with both methods for 72 h. Error bars represent the standard deviation of three tests.

CBP release was evaluated in 0.1 M PBS at pH 5, 6, and 7.4 to assess the influence of the pH and to mimic biological conditions. The supernatant sampled during the release process was analyzed using both the optimized DPV and the UV–Vis method. The cumulative release profiles obtained with both methods were represented (Figure 6) and compared (Figure 7).

The highest release was obtained at pH 5, followed by pH 6 and 7.4. A greater release at pH 5 proves advantageous in drug delivery applications, as tumor tissues are commonly recognized to have a more acidic pH compared to healthy tissues [38,39,40]. Comparative results regarding the release profiles are represented in Figure 7. An average correlation of 102.99 (RSD 5.22%) was obtained considering a 72 h release process. The minimal variance between the two techniques may be ascribed to variations in their sensitivity, with DPV demonstrating superior sensitivity. Higher discrepancies between the two methods were noticed at two specific time points (Figure 7). These can be explained by the higher standard deviation of the DPV method compared to the UV–Vis one as well as by the inter-electrode variability, due to the in-house screen-printed nature of the used electrodes. However, the overall cumulative release profile remains highly similar for the two methods, as demonstrated by ANOVA analysis.

**Figure 8:**
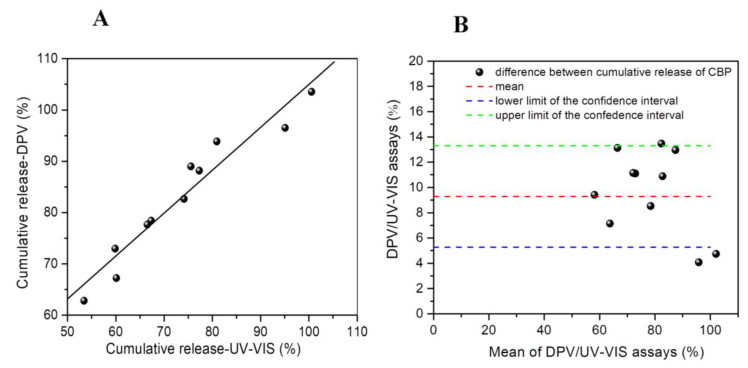
(**A**) The correlation between the measured cumulative release of CBP in 0.1 M PBS solution of pH 5 determined by DPV and the UV–Vis control method was investigated (*n* = 11). (**B**) Bland–Altman analysis was conducted to assess the agreement between the cumulative release of CBP obtained using the optimized electrochemical method and UV–Vis spectrophotometry for the release of CBP from nanosomes. The release was conducted in 0.1 M PBS of pH 5 and tested using both methods over a period of 72 h. The plotted points depict the differences between the cumulative release (%) calculated based on the data obtained from DPV and UV–Vis assays relative to the mean of the cumulative release values. The medium dotted horizontal red line represents the estimated mean bias of 9.29%, while the two dashed horizontal lines indicate the upper (green) and lower (blue) limits of the 95% confidence interval for agreement determined by the mean standard deviation (SD = ±4.01).

Cumulative release was evaluated at three different pH levels using both electrochemical and control methods, with three replicates per test, although statistical analysis was only conducted at pH 5 (Figure 8A). As evident from the presented data, there was a dose-dependent correlation observed, and the difference between the evaluated methods was not statistically significant, demonstrating an acceptable regression coefficient of 0.984 (*p* < 0.001) and a slope of 0.837.

The agreement of the results obtained for the cumulative release of CBP from the pharmaceutical product (nanosomes loaded with CBP) was assessed using the Bland–Altman plot (Figure 8B), comparing the DPV and UV–Vis assays. The Bland–Altman plot illustrates the differences between all datasets and the mean of the cumulative release obtained by both methods. The mean difference in cumulative release between the two procedures was 9.29%, with limits of agreement of 4.01 and 13.31 (Figure 8B), indicating strong agreement between the methods for determining CBP release in 0.1 M PBS pH 5. Approximately 95% of the differences fell within these limits. Thus, based on the Bland–Altman plot, it can be concluded that the two analytical methods exhibit good agreement and that the optimized electrochemical detection strategy demonstrates high accuracy and robustness. The strong correlation observed in the results underscores the suitability of the DPV method for monitoring CBP release from nanosomes.

## 4. Conclusions

In this study, in-house screen-printed carbon electrodes were developed for the detection of CBP from a variety of samples such pharmaceutical formulations and biological fluids. The in-house screen-printed surface demonstrated better performances compared to commercial screen-printed electrodes, indicating its applicability as a useful alternative for CBP detection from pharmaceutical formulations, especially in the early phases of formulation when numerous tests are required.

The influence of the pH and scan rate on the electrochemical behavior of CBP were studied, followed by method optimization. The optimized detection method was based on DPV and had a linear range between 5 and 500 μg/mL, with a detection limit of 1.6 µg/mL. This method successfully detected CBP loaded and released from lipid-based carriers. The release of CBP was monitored at different pH values and proved to be Ph-dependent, with higher release at a more acidic pH. UV–Vis spectrophotometry served as a control method, showing strong correlations with DPV results and affirming the electrochemical method’s suitability for CBP detection in pharmaceutical formulations. This highlights the potential of electrochemical methods as cost-effective, sensitive alternatives for quality control in pharmaceutical formulation development, offering improved sensitivity and lower costs while maintaining accuracy. Moreover, the applicability of the method for monitoring CBP in deproteinized serum, artificial saliva, and tears with good recoveries was also evaluated, serving as proof-of-concept for the detection of CBP from complex matrices.

## Figures and Tables

**Table 1:** Peak potentials and intensities for the oxidation/reduction in [Fe(CN)_6_]^3−/4−^ on different electrode surfaces.

Electrode Type	E_ox_ (V)	I_ox_ (µA)	E_red_ (V)	I_red_ (µA)
Carbon Dropsens^®^ electrode (d = 4 mm)	0.256	164	0.027	−191
In-house screen-printed carbon electrode (d = 4 mm)	0.542	209	−0.274	−208
In-house screen-printed carbon electrode, after activation (d = 4 mm)	0.252	402	0.040	−380

**Table 2:** Comparison of the present study with previously published data.

Electrode	Method	Linear Range (μM)	LOD (μM)	Matrix	Ref
Pt electrode	LSV, direct	50–1000	30	Cell culture supernatant	[27]
Pt electrode CPE	CV, direct	10–300	8	Aqueous solutions	[28]
GCE-DNA	DPV, indirect	5.7–40	5.7	Human serum	[29]
GCE-CNT-epoxy	DPV, indirect	0–100	0.014	Aqueous solutions	[30]
**SPCE**	**DPV, direct**	**13.5–1350**	**4.5**	**Loading and release buffers containing CBP** **Artificial saliva** **Artificial tears**	**This work**

LSV—linear sweep voltammetry; CPE—carbon paste electrode; CV—Cyclic voltammetry; GCE—glassy carbon electrode; DPV—differential pulse voltammetry; CNT—carbon nanotubes; SPCE—screen-printed carbon electrode. Results from this work presented in bold.

**Table 3:** Selectivity studies for the electrochemical detection of CBP on in-house screen-printed electrodes.

Sample	Concentration (µg/mL)	Recovery (%)	RSD (%)
CBP + Cisplatin	25	2.04	2.30
	100	-	-
	200	1.06	1.60
CBP + Dopamine	25	95.79	0.75
	100	101.68	3.51
	200	108.93	1.80
CBP + Ascorbic acid	25	104.51	1.43
	100	87.81	1.65
	200	100.01	1.87
CBP + Ca(NO_3_)_2_	25	99.56	1.17
	100	108.17	2.04
	200	98.87	1.22
CBP + Na_3_C_6_H_5_O_7_	25	113.55	1.24
	100	118.85	2.48
	200	101.33	0.87
CBP + MgCl_2_	25	118.49	0.67
	100	93.83	3.22
	200	112.70	0.71
CBP + Li_2_SO_4_	25	101.75	1.38
	100	103.22	2.23
	200	105.11	1.75
CBP + NH_4_Cl	25	109.61	1.33
	100	109.92	2.45
	200	108.78	1.72
CBP + MgBr_2_	25	141.54	0.79
	100	128.38	1.54
	200	107.89	1.89

**Table 4:** Real sample analysis.

Sample	Concentration (µg/mL)	Recovery (%)	RSD (%)
Deproteinized serum Dilution 1:10	25	103.72	1.42
	100	102.24	6.06
	200	92.74	8.97
Saliva Dilution 1:10	25	102.92	2.78
	100	106.31	3.27
	200	99.80	2.56
Artificial teras Dilution 1:10	25	96.94	3.31
	100	95.44	1.04
	200	110.05	1.61

**Table 5:** ANOVA statistical data obtained based on the recovery values of CBP estimated based on DPV tests.

Source of Variation	SS	Df	MS	F	*p*-Value	F Crit
Between Groups	26.7	1	26.7	2.71	0.279	3.28
Within Groups	383.4	54	44.51			
					*p*-value theoretical	
Total	410.1	55			0.05	

**Table 6:** Parameters obtained for the spectrophotometric analysis of CAR in different buffers.

Media	λ_max_ (nm)	Calibration Curve Equation	R^2^
0.1 M PBS pH 5.0	231	A = 0.007[CBP] − 0.027	0.999
0.1 M PBS pH 6.0	230	A = 0.007[CBP] + 0.0016	0.999
0.1 M PBS pH 7.4	231	A = 0.007[CBP] − 0.0298	0.999

**Table 7:** Comparison of the loading data obtained using the UV–Vis and the DPV methods.

Loading Solution	Quantification Method	EE (%)	LC (%)
2 mg/mL CBP in 0.1 M PBS pH 5	DPV	32.76	1.15
	UV–Vis	34.37	1.11

## Data Availability

Data is contained within the article. Dataset available upon request from the authors.
